# Supplementary material for: Effect of autologous amniotic membrane and fluid on wound healing and complications of cesarean section: Study protocol of a factorial randomized controlled trial
Source: PLoS One. 2025 Dec 19;20(12):e0337907. doi: 10.1371/journal.pone.0337907 (PMC12716681; doi:10.1371/journal.pone.0337907)
Supplement: S1 File — IRB’s ethical approval letter. (PDF) [file pone.0337907.s001.pdf]

Tehran University of Medical Sciences

School of Medicine- Tehran University of Medical Sciences

Research Ethics Committees Certificate

<table>
<tr><td>Approval ID:</td><td>IR.TUMS.MEDICINE.REC.1402.758</td><td rowspan="2">Approval Date:</td><td rowspan="2">2024-03-13</td></tr>
<tr><td>Evaluated by:</td><td>Research Ethics Committees of School of Medicine- Tehran University of Medical Sciences</td></tr>
<tr><td>Status:</td><td colspan="3">Approved</td></tr>
<tr><td>Approval Statement:</td><td colspan="3">The project was found to be in accordance to the ethical principles and the national norms and standards for conducting Medical Research in Iran.
Notice:
1. Although the proposal has been approved by the Biomedical Research Ethics Committee, meeting the professional and legal requirements is the sole responsibility of the PI and other project collaborators.
2. This certificate is reliant on the proposal/documents received by this committee on 2024-03-13. The committee must be notified by the PI as soon as the proposal/documents are modified.</td></tr>
<tr><td>Proposal Title:</td><td colspan="3">Investigating the effect of using autologous amniotic membranes fluid on wound healing and complications of cesarean surgery: a factorial randomized clinical trial</td></tr>
<tr><td>Principal Investigator:</td><td colspan="3">Name: Amene Abiri
Email: abiri@sina.tums.ac.ir</td></tr>
</table>

Dr. Nima Rezaei
Committee Director
School of Medicine- Tehran University of Medical Sciences

Dr. Mahshad Khodarahmian
Committee Secretary
School of Medicine- Tehran University of Medical Sciences
