# Supplementary material for: Effect of autologous amniotic membrane and fluid on wound healing and complications of cesarean section: Study protocol of a factorial randomized controlled trial
Source: PLoS One. 2025 Dec 19;20(12):e0337907. doi: 10.1371/journal.pone.0337907 (PMC12716681; doi:10.1371/journal.pone.0337907)
Supplement: S3 File — Proposal file in Farsi, which was approved by the IRB. (DOCX) [file pone.0337907.s003.docx]

**عنوان طرح تحقیقاتی :** بررسی تاثیر استفاده از پرده و مایع آمنیون اوتولوگ بر روی ترمیم و عوارض زخم جراحی سزارین: کارآزمایی بالینی تصادفی شده فاکتوریال

**Title :** Investigating the effect of using autologous amniotic membranes fluid on wound healing and complications of cesarean surgery: a factorial randomized clinical trial

**کد رهگیری:** ۶۹۷۰۶

**پژوهشگر :** آمنه عبیری

**تخصص:** فلوشیپ پریناتولوژی

**کد طرح:** ۱۴۰۲-۴-۴۱۸-۶۹۷۰۶

**کد اخلاق:** IR.TUMS.MEDICINE.REC.1402.758

**تاریخ ثبت اولیه:** ۱۴۰۲/۰۹/۲۵ ۱۱:۵۶:۲۲

**تاریخ ارسال:** ۱۴۰۲/۱۰/۱۳ ۱۳:۳۴:۵۹

**تاریخ این ویراست:** ۱۴۰۲/۱۲/۰۵ ۱۴:۳۳:۵۱

**مرکز هدف اول:** دانشکده پزشکی/بیماری های زنان و زایمان (بیمارستان آرش)

**مرکز هدف دوم:** پژوهشکده سلامت خانواده/م ت. مادر، جنین و نوزاد

مشخصات کلی و چکیده طرح

**عنوان فارسی طرح**

بررسی تاثیر استفاده از پرده و مایع آمنیون اوتولوگ بر روی ترمیم و عوارض زخم جراحی سزارین: کارآزمایی بالینی تصادفی شده فاکتوریال

**عنوان انگلیسی طرح**

Investigating the effect of using autologous amniotic membranes fluid on wound healing and complications of cesarean surgery: a factorial randomized clinical trial

**کلید واژه ها**

ترمیم و عوارض زخم جراحی سزارین، پرده و مایع آمنیون اتولوگ

**نوع مطالعه**

كارآزمایی بالینی تصادفی

**خلاصه ضرورت اجرای طرح**

پرده و مایع آمنیوتیک انسان از اوایل قرن گذشته برای التیام زخم ها (حاد و مزمن) و سوختگی ها استفاده شده است. همانطور که قبلا تایید شده، استفاده از این محصول می تواند زمان بهبودی، عفونت و درد را کاهش دهد (۱۰). با توجه به یافته ها، پرده آمنیون قادر به القای جذب سلول های اندوتلیال و آنژیوژنز (رگ زایی) است. در یک متاآنالیز اخیر، نشان داده شد که بیماران مبتلا به زخم های مزمن اندام تحتانی تحت درمان با پرده ی آمنیون در مقایسه با گروه پانسمان مراقبت استاندارد در طول یک دوره ی ۶ هفته ای، میزان موفقیت قابل توجهی بالاتری در درمان خود داشتند (۱۱). همچنین، مشخص شده است که پرده ی آمنیون زنده انجماد شده انسانی (hCVAM) می تواند رشد باکتری ها را در زخم ها مهار کند. به نظر می رسد این به دلیل دو پپتید ضد میکروبی به نام های HBD۲ و HBD۳ باشد.

**خلاصه روش اجرا و شیوه های تحلیل**

مطالعه به صورت آینده نگر و مداخله ای، کارآزمایی بالینی تصادفی شده، شاهد دار ، فاکتوریال و دو سو کور می باشد. این مطالعه در بیمارستان جامع بانوان آرش در بین زنان باردار مراجعه کننده به بیمارستان جامع بانوان آرش که جهت سزارین مراجعه دارند انجام خواهد شد. گروه های مداخله شامل گروه های : گروه مداخله A: دریافت پانسمان با پرده آمنیون اوتولوگ زخم جراحی سزارین
گروه مداخله B: دریافت پانسمان با اسپری مایع آمنیون اوتولوگ زخم جراحی سزارین
گروه مداخله C: دریافت پانسمان و پرده آمنیون اوتولوگ و اسپری مایع آمنیون اوتولوگ زخم جراحی سزارین
گروه شاهد D: دریافت پانسمان زخم جراحی سزارین
خواهد بود. مایع آمنیون از خود بیمار دریافت شده و در هر مرحله ی بستن زخم اسپری خواهد شد. پرده ی آمنیون هم از خود بیمار دریافت شده و بر روی زخم قرار داده خواهد شد. بیماران در روز دوم، دهم و بیست و هشتم از نظر وجود پیامدها بررسی خواهند شد.

**آیا این طرح جزء طرح های مشترک بین المللی می باشد؟**

خیر

**مقدمه-بیان مساله**

جراحی سزارین مطمئنا از زمانی که توسط James Munro Kerr در اوایل قرن بیستم میلادی با تکنیک‌ بهینه‌تری معرفی و فراگیر شد، جان انسان‌های بسیاری را حفظ کرده است (۱). با افزایش روز افزون تعداد جراحی‌های سزارین مشاهده‌ی مقالاتی که از این پدیده به عنوان یک اپیدمی یاد می‌کنند بسیار متداول گشته است. موارد انجام عمل سزارین طی سال‌های ۱۹۹۰ تا ۲۰۱۴ یک افزایش ۴/۱۲ درصدی (از ۷/۶ تا ۱/۱۹ درصد) در سطح جهانی از خود نشان داده است (۲). شیوع ۴۸ درصدی سزارین در ایران نیز بسیار بالاتر از توصیه‌ی ۱۵ درصدی سازمان جهانی بهداشت می باشد (۳)، بنابراین لزوم توجه به عوارض جراحی سزارین به عنوان پدیده‌ای رایج در مادران ایرانی بیشتر از سایر کشورها می باشد.
دلایل افزایش تمایل زنان ایرانی به انجام سزارین را می‌توان به سه دسته تقسیم کرد: عوامل اجتماعی و دموگرافیک، عوامل مامایی-بالینی و عوامل غیرمامایی-غیربالینی (۴). سطح تحصیلات بالاتر و سن بالای بارداری به عنوان مهم‌ترین عوامل اجتماعی و دموگرافیک تمایل به سزارین شناخته شده‌اند، همچنین سزارین قبلی و زجرجنینی مهم‌ترین عوامل مامایی-بالینی تمایل به سزارین می باشند. در نهایت مهم‌ترین عوامل غیرمامایی-غیربالینی تمایل به سزارین بین زنان ایرانی ترس از زایمان واژینال و توصیه‌ی پزشک می باشد (۴). سزارین مزایای بسیاری برای مادر دارد که می‌توان از حفاظت از پرینه به عنوان غالب‌ترین مشوق بیولوژیکی سزارین نام برد. علاوه بر مزایای بیولوژیکی سزارین در پیشگیری از عوارضی چون بی‌اختیاری ادراری در کوتاه مدت، می‌توان رفع ترس از درد (توکوفوبیا) و هم‌چنین برطرف شدن نیاز به داشتن اختیار و کنترل بر روی فرایند زایمان را از جمله مزایای روان‌شناختی سزارین نامید. سایر مزایای سزارین شامل زمان بهبودی کوتاه‌تر، مدت زمان بستری کم‌تر و میزان بالای تغذیه‌ی پستانی می‌باشد (۵).
با اینکه سزارین مزایای زیادی دارد و جان‌های بسیاری را حفظ کرده است، اما همچنان عوارض خطرناک این عمل می تواند جان و سلامت مادرانی را که تحت جراحی سزارین قرار می‌گیرند را تهدید ‌کند. همانند سایر جراحی‌ها، سزارین نیز می‌تواند با عفونت محل جراحی همراه باشد. این عفونت می‌تواند صرفا به زخم محدود باشد یا این که پیشرفته‌تر بوده و باعث اندومتریت شود. عوارض زخم سزارین شامل عفونت سطحی، و جمع‌ شدن مایعات مانند سروما یا هماتوم است که ۳ تا ۱۵ درصد موارد را تحت تاثیر قرار می‌دهد (۶, ۷).
پانسمان محل جراحی یکی از مهم ترین عوامل تعیین کننده ی روند بهبود زخم است. درمان زخم با فشار منفی (NPWT) یک گزینه درمانی برای تسریع بهبود زخم به ویژه در مورد زخم های باز است. در یک مرور سیستماتیک و متاآنالیز، نشان داده شد که NPWT پیشگیرانه می تواند به طور قابل توجهی خطر ابتلا به عفونت محل جراحی را از ۱۲.۵ به ۵.۲ کاهش دهد و در نتیجه در مقایسه با پانسمان زخم کلاسیک، ۵۸ کاهش یابد (۸). با این حال، همانطور که گفته شد، این روش بسیار گران است. در یک مطالعه تحلیلی-تصمیم گیری در مورد ارزیابی هزینه-فایده NPWT برای زخم های سزارین، نشان داده شده است که بیماران با ریسک پایین عفونت محل جراحی برای NPWT مناسب نیستند. از سوی دیگر، برای گروه پرخطر (دیابتی‌ها، مبتلایان به سرکوب سیستم ایمنی و BMI ≥ ۳۰)، NPWT یک روش مقرون ‌به ‌صرفه برای پیشگیری از عفونت محل جراحی و عوارض آن نیست (۹).
پرده آمنیوتیک انسان از اوایل قرن گذشته برای التیام زخم ها (حاد و مزمن) و سوختگی ها استفاده شده است. همانطور که قبلا تایید شده، استفاده از این محصول می تواند زمان بهبودی، عفونت و درد را کاهش دهد (۱۰). با توجه به یافته ها، پرده آمنیون قادر به القای جذب سلول های اندوتلیال و آنژیوژنز (رگ زایی) است. در یک متاآنالیز اخیر، نشان داده شد که بیماران مبتلا به زخم های مزمن اندام تحتانی تحت درمان با پرده ی آمنیون در مقایسه با گروه پانسمان مراقبت استاندارد در طول یک دوره ی ۶ هفته ای، میزان موفقیت قابل توجهی بالاتری در درمان خود داشتند (۱۱). همچنین، مشخص شده است که پرده ی آمنیون زنده انجماد شده انسانی (hCVAM) می تواند رشد باکتری ها را در زخم ها مهار کند. به نظر می رسد این به دلیل دو پپتید ضد میکروبی به نام های HBD۲ و HBD۳ باشد (۱۲).
در حال حاضر پرده آمنیون به صورت فریز شده جهت استفاده در پانسمان زخم ها موجود می باشد. با توجه به مباحث مطرح شده در بالا و مزایای استفاده از پیوند اتولوگ همانند کاهش پاسخ ایمنی به پیوند، کاهش احتمال انتقال بیماری از طریق پیوند، نتایج بهتر پیوندهای اتولوگ، دسترسی راحت تر، هزینه کمتر و مشکلات اخلاقی کمتر این مطالعه جهت بررسی تاثیر استفاده از پرده و مایع آمنیون خود بیمار بر روی ترمیم و عوارض زخم سزارین انجام خواهد شد.

**بررسی متون**

• محسنی و همکاران (۱۳) در پژوهشی با عنوان «پرده آمنیون برای کنترل درد پس از سزارین» به بررسی ارتباط استفاده از پرده ی آمنیون خود بیمار بر روی درد محل جراحی پرداخته اند. در این مطالعه که به صورت کارآزمایی بالینی تصادفی شده انجام شده دو گروه ۴۵ نفره انجام شد، یک گروه پانسمان ساده ی متداول را دریافت کردند و گروه دوم پانسمان با پرده آمنیون را نیز دریافت کرد. میزان درد در هر دو گروه با استفاده از visual analogue scale (VAS) سنجیده شد. همچنین نیاز به دریافت مسکن در هر دو گروه طی ۲۴ ساعت پس از جراحی سنجیده شد. طبق یافته های این مطالعه استفاده از پانسمان پرده آمنیون می تواند در کاهش درد بعد از سزارین موثر باشد و نیاز بیماران را به مسکن برطرف کند. از این رو می توان از آن به عنوان یک روش مکمل موثر در کنار مسکن های معمول برای تسکین درد استفاده کرد.
• ملازم و همکاران (۱۴) در مقاله ی «تاثیر پرده آمنیون روی ترمیم زخم جراحی سزارین: یک مطالعه کارآزمایی بالینی» با استفاده از یک متدولوژی دوسو کور کارآزمایی بالینی به بررسی اثر پرده آمنیون روی ترمیم زخم با استفاده از پرسشنامه REEDA پرداخته است. یافته ‌های این مطالعه که در هر بازوی کارآزمایی ۴۵ نفر حضور داشته است، نشان داد که استفاده از پانسمان پرده آمنیوتیک می‌تواند در ترمیم زخم در مراحل اولیه سزارین مفید باشد.
• سرنا و همکاران (۱۵) در مقاله ی «کارآزمایی بالینی تصادفی شده ی استفاده از پرده آمنیون فریز شده روی زخم پای دیابتی» به بررسی ۷۶ بیمار با زخم پای دیابتی پرداخته اند. این کارآزمایی بالینی تصادفی شده نشان داد که در افرادی که پرده آمنیون را دریافت کرده بودند نسبت به افرادی که پانسمان متداول انجام شده بود در هفته های ۱۲ و ۱۶ احتمال بسته شدند زخم بسیار بیشتر بوده.

**اهداف کلی , اختصاصی و کاربردی**

اهداف اصلی طرح:
بررسی تاثیر استفاده از پرده آمنیون بر روی ترمیم و عوارض زخم جراحی سزارین
بررسی تاثیر استفاده از مایع آمنیون اسپری شده بر روی ترمیم و عوارض زخم جراحی سزارین
بررسی تاثیر استفاده همزمان از پرده آمنیون و مایع آمنیون اسپری شده بر روی ترمیم و عوارض زخم جراحی سزارین
مقایسه تاثیر استفاده از پرده آمنیون، مایع آمنیون اسپری شده و استفاده ی همزمان این دو بر روی ترمیم و عوارض زخم جراحی سزارین

اهداف فرعی طرح:
۱. بررسی تاثیر استفاده از پرده آمنیون بر روی ترمیم زخم جراحی سزارین با استفاده از معیار POSAS در روزهای 10 و ۲۸ پس از جراحی
۲. بررسی تاثیر استفاده از پرده آمنیون بر روی بروز عفونت محل جراحی سزارین
۳. بررسی تاثیر استفاده از پرده آمنیون بر روی دهیسنس زخم جراحی سزارین
۴. بررسی تاثیر استفاده از پرده آمنیون بر روی تشکیل هماتوم در زخم جراحی سزارین
۵. بررسی تاثیر استفاده از پرده آمنیون بر روی تشکیل سروما در زخم جراحی سزارین
۶. بررسی تاثیر استفاده از پرده آمنیون بر روی درد در زخم جراحی سزارین در ساعت های ۴، ۱۲، ۲۴، ۳۶، پس از جراحی
۷. بررسی تاثیر استفاده از پرده آمنیون بر روی دفعات دریافت مسکن تزریقی در 36 ساعت اول پس از جراحی
۸. بررسی تاثیر استفاده از پرده آمنیون بر بروز هرگونه عوارض جانبی
۹. بررسی تاثیر استفاده از پرده آمنیون بر مدت زمان بستری در بیمارستان
۱۰. بررسی تاثیر استفاده از مایع آمنیون اسپری شده بر روی ترمیم زخم جراحی سزارین با استفاده از معیار POSAS در روزهای ۷ و ۲۸ پس از جراحی
۱۱. بررسی تاثیر استفاده از مایع آمنیون اسپری شده بر روی بروز عفونت محل جراحی سزارین
۱۲. بررسی تاثیر استفاده از مایع آمنیون اسپری شده بر روی دهیسنس زخم جراحی سزارین
۱۳. بررسی تاثیر استفاده از مایع آمنیون اسپری شده بر روی تشکیل هماتوم در زخم جراحی سزارین
۱۴. بررسی تاثیر استفاده از مایع آمنیون اسپری شده بر روی تشکیل سروما در زخم جراحی سزارین
۱۵. بررسی تاثیر استفاده از مایع آمنیون اسپری شده بر روی درد در زخم جراحی سزارین در ساعت های ۴، ۱۲، ۲۴، ۳۶، پس از جراحی
۱۶. بررسی تاثیر استفاده از مایع آمنیون اسپری شده بر روی دفعات دریافت مسکن تزریقی در 36 ساعت اول پس از جراحی
۱۷. بررسی تاثیر استفاده از مایع آمنیون اسپری شده بر بروز هرگونه عوارض جانبی
۱۸. بررسی تاثیر استفاده از مایع آمنیون اسپری شده بر مدت زمان بستری در بیمارستان
۱۹. بررسی تاثیر استفاده همزمان از پرده آمنیون و مایع آمنیون اسپری شده بر روی ترمیم زخم جراحی سزارین با استفاده از معیار POSAS در روزهای 10 و ۲۸ پس از جراحی
۲۰. بررسی تاثیر استفاده همزمان از پرده آمنیون و مایع آمنیون اسپری شده بر روی بروز عفونت محل جراحی سزارین
۲۱. بررسی تاثیر استفاده همزمان از پرده آمنیون و مایع آمنیون اسپری شده بر روی دهیسنس زخم جراحی سزارین
۲۲. بررسی تاثیر استفاده همزمان از پرده آمنیون و مایع آمنیون اسپری شده بر روی تشکیل هماتوم در زخم جراحی سزارین
۲۳. بررسی تاثیر استفاده همزمان از پرده آمنیون و مایع آمنیون اسپری شده بر روی تشکیل سروما در زخم جراحی سزارین
۲۴. بررسی تاثیر استفاده همزمان از پرده آمنیون و مایع آمنیون اسپری شده بر روی درد در زخم جراحی سزارین در ساعت های ۴، ۱۲، ۲۴، ۳۶، پس از جراحی
۲۵. بررسی تاثیر استفاده همزمان از پرده آمنیون و مایع آمنیون اسپری شده بر روی دفعات دریافت مسکن تزریقی در 36 ساعت اول پس از جراحی
۲۶. بررسی تاثیر استفاده همزمان از پرده آمنیون و مایع آمنیون اسپری شده بر بروز هرگونه عوارض جانبی
۲۷. بررسی تاثیر استفاده همزمان از پرده آمنیون و مایع آمنیون اسپری شده بر مدت زمان بستری در بیمارستان
۲۸. مقایسه تاثیر استفاده از پرده آمنیون و مایع آمنیون اسپری شده بر روی ترمیم زخم جراحی سزارین با استفاده از معیار POSAS در روزهای 10 و ۲۸ پس از جراحی
۲۹. مقایسه تاثیر استفاده از پرده آمنیون، مایع آمنیون اسپری شده و استفاده همزمان این دو بر روی بروز عفونت محل جراحی سزارین
۳۰. مقایسه تاثیر استفاده از پرده آمنیون، مایع آمنیون اسپری شده و استفاده همزمان این دو بر روی دهیسنس زخم جراحی سزارین
۳۱. مقایسه تاثیر استفاده از پرده آمنیون، مایع آمنیون اسپری شده و استفاده همزمان این دو بر روی تشکیل هماتوم در زخم جراحی سزارین
۳۲. مقایسه تاثیر استفاده از پرده آمنیون، مایع آمنیون اسپری شده و استفاده همزمان این دو بر روی تشکیل سروما در زخم جراحی سزارین
۳۳. مقایسه تاثیر استفاده از پرده آمنیون، مایع آمنیون اسپری شده و استفاده همزمان این دو بر روی درد در زخم جراحی سزارین در ساعت های ۴، ۱۲، ۲۴، ۳۶، پس از جراحی
۳۴. مقایسه تاثیر استفاده از پرده آمنیون، مایع آمنیون اسپری شده و استفاده همزمان این دو بر روی دفعات دریافت مسکن تزریقی در 36 ساعت اول پس از جراحی
۳۵. مقایسه تاثیر استفاده از پرده آمنیون، مایع آمنیون اسپری شده و استفاده همزمان این دو بر بروز هرگونه عوارض جانبی
۳۶. مقایسه تاثیر استفاده از پرده آمنیون، مایع آمنیون اسپری شده و استفاده همزمان این دو بر مدت زمان بستری در بیمارستان

اهدف کاربردی طرح:
استفاده از پانسمان پرده آمنیون و اسپری مایع آمنیون می تواند باعث کاهش عوارض شایع زخم جراحی سزارین (درد، عفونت، هماتوم و اسکار) شود. با توجه به شیوع بالای سزارین بین مادران ایرانی هر گونه کاهش عوارض این جراحی بسیار مهم بوده و سلامت مادر، نوزاد، خانواده و جامعه را افزایش می دهد.

**فهرست منابع مورد استفاده**

۱. Todman D. A history of caesarean section: from ancient world to the modern era. Australian and New Zealand Journal of Obstetrics Gynaecology. ۲۰۰۷;۴۷(۵):۳۵۷-۶۱.
۲. Betrán AP, Ye J, Moller A-B, Zhang J, Gülmezoglu AM, Torloni MR. The increasing trend in caesarean section rates: global, regional and national estimates: ۱۹۹۰-۲۰۱۴. PloS one. ۲۰۱۶;۱۱(۲):e۰۱۴۸۳۴۳.
۳. Rafiei M, Ghare MS, Akbari M, Kiani F, Sayehmiri F, Sayehmiri K, et al. Prevalence, causes, and complications of cesarean delivery in Iran: A systematic review and meta-analysis. International journal of reproductive biomedicine. ۲۰۱۸;۱۶(۴):۲۲۱.
۴. Azami-Aghdash S, Ghojazadeh M, Dehdilani N, Mohammadi M. Prevalence and causes of cesarean section in Iran: systematic review and meta-analysis. Iranian journal of public health. ۲۰۱۴;۴۳(۵):۵۴۵.
۵. Gregory KD, Jackson S, Korst L, Fridman M. Cesarean versus vaginal delivery: whose risks? Whose benefits? American journal of perinatology. ۲۰۱۲;۲۹(۰۱):۰۷-۱۸.
۶. Temming LA, Raghuraman N, Carter EB, Stout MJ, Rampersad RM, Macones GA, et al. Impact of evidence-based interventions on wound complications after cesarean delivery. American journal of obstetrics gynecology. ۲۰۱۷;۲۱۷(۴):۴۴۹. e۱-. e۹.
۷. Conner SN, Verticchio JC, Tuuli MG, Odibo AO, Macones GA, Cahill AG. Maternal obesity and risk of postcesarean wound complications. American journal of perinatology. ۲۰۱۳:۲۹۹-۳۰۴.
۸. Strugala V, Martin R. Meta-Analysis of Comparative Trials Evaluating a Prophylactic Single-Use Negative Pressure Wound Therapy System for the Prevention of Surgical Site Complications. Surgical Infections. ۲۰۱۷;۱۸(۷):۸۱۰-۹.
۹. Shea SK, Soper DE. Prevention of Cesarean Delivery Surgical Site Infections. Obstetrical & Gynecological Survey. ۲۰۱۹;۷۴(۲):۹۹-۱۱۰.
۱۰. McKenna B, Summers NJ. Amnion: The Ideal Scaffold for Treating Full-Thickness Wounds of the Lower Extremity. Clinics in Podiatric Medicine and Surgery. ۲۰۱۸;۳۵(۱):۱-۹.
۱۱. Haugh AM, Witt JG, Hauch A, Darden M, Parker G, Ellsworth WA, et al. Amnion membrane in diabetic foot wounds: a meta-analysis. Plastic and Reconstructive Surgery Global Open. ۲۰۱۷;۵(۴).
۱۲. Mao Y, Hoffman T, Singh-Varma A, Duan-Arnold Y, Moorman M, Danilkovitch A, et al. Antimicrobial peptides secreted from human cryopreserved viable amniotic membrane contribute to its antibacterial activity. Scientific reports. ۲۰۱۷;۷(۱):۱۳۷۲۲.
۱۳. Mohseni F, Saem J, Sekhavati E, Molazem Z, Tabrizi R. Amniotic Membrane for Pain Control After Cesarean Section. Crescent Journal of Medical & Biological Sciences. ۲۰۱۸;۵(۳).
۱۴. Molazem Z, Mohseni F, Rakhshan M, Keshavarzi S, Younesi M. The effect of amniotic membrane on the healing of cesarean wounds: a randomized clinical trial. Women’s Health Bulletin. ۲۰۱۸;۵(۲):۱-۶.
۱۵. Serena TE, Yaakov R, Moore S, Cole W, Coe S, Snyder R, et al. A randomized controlled clinical trial of a hypothermically stored amniotic membrane for use in diabetic foot ulcers. Journal of Comparative Effectiveness Research. ۲۰۲۰;۹(۱):۲۳-۳۴.
۱۶. Cromi A, Ghezzi F, Gottardi A, Cherubino M, Uccella S, Valdatta L. Cosmetic outcomes of various skin closure methods following cesarean delivery: a randomized trial. American journal of obstetrics and gynecology. ۲۰۱۰;۲۰۳(۱):۳۶-e۱.
۱۷. Draaijers LJ, Tempelman FRH, Botman YAM, Tuinebreijer WE, Middelkoop E, Kreis RW, et al. The Patient and Observer Scar Assessment Scale: A Reliable and Feasible Tool for Scar Evaluation. Plastic and Reconstructive Surgery. ۲۰۰۴;۱۱۳(۷).
۱۸. Shao K, Parker JC, Taylor L, Mitra N, Sobanko JF. Reliability of the patient and observer scar assessment scale when used with postsurgical scar photographs. Dermatologic surgery. ۲۰۱۸;۴۴(۱۲):۱۶۵۰.

مجریان و همکاران

| **نام مجری/ همکار** | **نام خانوادگی مجری/ همکار** | **نام انگلیسی** | **نام خانوادگی انگلیسی** | **محل کار** | **پست الکترونیک** | **نوع همکاری در این طرح** | **تخصص** |
| --- | --- | --- | --- | --- | --- | --- | --- |
| مرضیه | وحید دستجردی | marzieh | Vahiddastjerdi | دانشکده پزشکی/بیماری های زنان و زایمان (بیمارستان آرش) | mvahid@tums.ac.ir | همکار | زنان و زایمان |
| آمنه | عبیری | amene | abiri | دانشکده پزشکی/بیماری های زنان و زایمان (بیمارستان آرش) | abiri@sina.tums.ac.ir | مجری | فلوشیپ پریناتولوژی |
| کسری | جعفری | kasra | jafari | خارج از دانشگاه علوم پزشکی تهران | kasra.e.jafari@gmail.com | همکار | اپیدمیولوژی |

روش اجرای مطالعه

**روش اجرا**

این مطالعه به صورت آینده نگر و مداخله ای می باشد
نوع مطالعه:
کارآزمایی بالینی تصادفی شده، فاکتوریال، شاهددار و دو سو کور
محل انجام مطالعه:
بیمارستان جامع بانوان آرش
جامعه پژوهش:
زنان باردار مراجعه کننده به بیمارستان جامع بانوان آرش جهت انجام سزارین
معیار ورود:
• سن ۱۸-45 سال
• حاملگی تک قلو
• برنامه ریزی برای زایمان سزارین
• سن حاملگی برنامه ریزی شده حداقل ۳۶ هفته در زمان زایمان
• هموگلوبین بالای 10g/dL
• قادر به ارائه رضایت آگاهانه
• قصد در دسترس بودن برای کل دوره پژوهشی و تکمیل تمام مراحل مطالعه مرتبط، از جمله مراجعات مطالعاتی بعدی و تماس های تلفنی
معیار عدم ورود:
• عدم تمایل به شرکت در مطالعه
• BMI بیشتر یا مساوی ۴۰ در زمان ورود به مطالعه
• جفت سرراهی یا چسبندگی جفت (Placenta previa or placenta accreta)
• جراحی قبلی روده یا اورولوژی
• حاملگی چند قلو
• اختلال شناخته شده یا مشکوک در عملکرد ایمونولوژیک از جمله عفونت با HIV، هپاتیت B یا C
• مصرف داروی سرکوب کننده ی ایمنی
• مصرف شناخته شده تنباکو یا مواد مخدر
• هر شرایطی که به نظر محقق ممکن است خطری برای سلامتی شرکت کننده ایجاد کند یا در ارزیابی اهداف مطالعه اختلال ایجاد کند.
• سابقه تشکیل کلوئید
• کوریوآمنیونیت یا سایر عفونتهای سیستمیک در زمان مراجعه برای سزارین، از جمله شواهد عفونت پوست زیر شکم (مانند قارچی و غیره)
• نیاز به سزارین اورژانسی (مثالهایی شامل، اما نه محدود به: زجر جنینی، جدا شدن جفت، پره اکلامپسی شدید یا اکلامپسی)
• پارگی پرده ها قبل از شروع جراحی
• مایع آمنیوتیک آغشته به مکونیوم یا آغشته به خون
• معاینه های واژینال متعدد
• پره اکلامپسی با علایم شدید
مداخله:
گروه مداخله A: دریافت پانسمان با پرده آمنیون اوتولوگ زخم جراحی سزارین
گروه مداخله B: دریافت پانسمان با اسپری مایع آمنیون اوتولوگ زخم جراحی سزارین
گروه مداخله C: دریافت پانسمان و پرده آمنیون اوتولوگ و اسپری مایع آمنیون اوتولوگ زخم جراحی سزارین
گروه شاهد D: دریافت پانسمان زخم جراحی سزارین
برای تمامی بیمارانی که در گروه مداخله A قرار می گیرند، پس از اتمامی جراحی، قسمتی از پرده آمنیون خودشان متناسب اندازه ی برش جراحی برداشته شده و قبل از اعمال پانسمان متداول با سرم نرمال سالین استریل شستشو داده شده و روی برش قرار داده می شود. پرده آمنیون اوتولوگ در تمام مدت بین زمان برداشت تا استفاده داخل رسیور استریل که با نرمال سالین استریل پر شده است، نگهداری خواهد شد. در گروه مداخله B مایع آمنیون اوتولوگ استخراج شده اسپری خواهد شد (نحوه ی استخراج مایع آمنیوتیک و اسپری کردن آن در ادامه توضیح داده خواهد شد). در گروه C هر دو مداخله انجام خواهد شد. در گروه شاهد D بیماران پانسمان متداول (standard of care) را دریافت خواهند کرد.
در طول جراحی متغیرهای زیر اندازه گیری خواهند شد:
زمان شروع عمل (زمان برش)
زمان برش رحم
توصیف مایع آمنیوتیک (رنگ، وجود یا عدم وجود راس، خون یا مکونیوم)
زمانی که بسته شدن رحم کامل می شود
زمان پایان جراحی
خونریزی تخمینی
نوع بسته شدن پوست
پس از انجام برش رحم مراحل زیر انجام می شود:
۱. پس از برش بخش تحتانی رحم، کیسه آمنیوتیک با سوراخ کننده آمنیوتیک (amniotic perforator) باز می شود.
۲. از سرنگ 10 میلی لیتری برای کشیدن مایع آمنیوتیک هنگام خروج از حفره آمنیوتیک استفاده خواهد شد.
۳. سپس سرنگ ۱۰ میلی لیتری حاوی مایع آمنیوتیک از نظر آلودگی (به عنوان مثال وجود مکونیوم) مشاهده می شود و سپس به دستگاه اسپری متصل می شود.
4. سپس مایع آمنیوتیک در سراسر برش بسته رحم و بخش تحتانی رحم اسپری می شود.
5. سپس فاسیا بسته می شود و برش فاسیال اسپری می شود (۱.۵ تا ۲.۰ میلی لیتر).
6. در نهایت بافت زیر جلدی بسته و اسپری می شود (۱.۵ تا ۲.۰ میلی لیتر) و سپس پوست بسته و اسپری می شود (۱.۵ تا ۲.۰ میلی لیتر).
7. سپس برش به صورت متداول پانسمان می شود.
پیگیری:
۱- ویزیت روز ترخیص:
شرکت کننده مراقبت های معمول بعد از عمل را در طول دوره بلافاصله پس از عمل دریافت خواهد کرد. اکثر بیماران سزارین در روز ۲ یا ۳ بعد از عمل مرخص می شوند. فعالیت های مطالعاتی زیر در این ویزیت انجام می شود:
• ارزیابی محل برش که شامل قرمزی، تورم، سفتی و همچنین درد در حالت عادی و با فشار ملایم است.
• میزان مصرف مسکن از زمان زایمان (ضد التهابی غیر استروئیدی و مخدرها).
• برنامه ریزی اولین ویزیت پیگیری که 12 تا ۸ روز پس از جراحی انجام می شود.
• تیم مطالعه سوابق پزشکی را برای علائم حیاتی زیر نظر گرفته و هرگونه شواهدی مبنی بر تب بعد از عمل (دمای مادر بیش از ۳۸ درجه سانتیگراد منبع آن و اقدامات انجام شده توسط تیم مراقبت) را ثبت خواهد کرد.
• مستندسازی هرگونه رویداد نامطلوب
۲- ویزیت روز هفتم:
افراد در بیمارستان جامع بانوان آرش تقریباً یک هفته پس از عمل او (روز 12-۸ پس از عمل) مشاهده خواهند شد. در این بازدید مراحل مطالعه زیر انجام خواهد شد:
• ارزیابی زخم.
• مستندسازی هرگونه علائم یا نشانه های عفونت.
• مستندسازی هرگونه عوارض جانبی.
• برنامه ریزی ویزیت مطالعاتی بعدی (تقریباً ۴ هفته پس از سزارین).
۳- ویزیت هفته چهارم:
افراد در بیمارستان جامع بانوان آرش تقریباً یک هفته پس از عمل او (هفته چهارم پس از عمل) مشاهده خواهند شد. در این بازدید مراحل مطالعه زیر انجام خواهد شد:
• ارزیابی زخم.
• مستندسازی هرگونه علائم یا نشانه های عفونت.
• مستندسازی هرگونه عوارض جانبی.
روش تصادفی سازی:
برای اطمینان از اینکه تعداد یکسانی از شرکت‌کنندگان به هر چهار گروه اختصاص داده می‌شوند، از تصادفی‌سازی بلوکی با اندازه بلوک‌های 4، 8 و 12 استفاده می‌شود. یک لیست تصادفی با استفاده از وب‌سایت Sealed Envelope (22) ایجاد می‌شود. تعداد بلوک‌ها و توالی افراد در لیست تصادفی فقط برای یک ناظر خارجی که مسئول تخصیص شرکت‌کنندگان است، در دسترس است و محققان از آن آگاه نیستند. این ناظر خارجی نقشی در تجزیه و تحلیل داده‌ها، ارزیابی و سنجش شرکت‌کنندگان نخواهد داشت. پایبندی به پروتکل تصادفی‌سازی در طول کارآزمایی به دقت مورد نظارت قرار خواهد گرفت.

روش کورسازی:
پانسمان هر 4 گروه مشابه هم بوده و با توجه به این که پرده آمنیون زیر پانسمان قرار می گیرد، بیمار از تخصیص خود خبردار نخواهد بود. کورسازی تیم جراحی مقدور نیست. پزشکی که وضعیت زخم را بررسی می کند از گروه فرد خبردار نخواهد بود و پانسمان بیمار قبل از بررسی توسط پزشک، توسط یک پزشک یا پرستار دیگر باز خواهد شد تا پزشک بررسی کننده از تخصیص فرد اطلاعی نداشته باشد. تحلیل گر آماری و مصاحبه کننده ای که با بیمار در طول دوره ی پیگیری تماس تلفنی خواهد داشت، از تخصیص وی اطلاعی نخواهند داشت. در مجموع به جز تیم جراحی و ناظر خارجی مسئول تخصیص تصادفی همه ی افراد دخیل در مطالعه کورسازی خواهند شد.
فرآیند شکستن کورسازی:
فرآیند شکستن کد تنها در صورت ضرورت و بروز عارضه جانبی جدی ( با تشخیص و دستور مجری / مجری همکار مطالعه) انجام خواهد شد. شکستن کورسازی مطالعه فقط در مواردی که بدون دانستن نوع درمان امکان مدیریت و درمان شرکت کننده بطور مناسب و کافی وجود نداشته باشد کد شکسته خواهد شد. گروه تخصیص هر کد، در پاکت در بسته در محل مطالعه قرار داده شده است و در صورت نیاز به شکستن کد در مواقع ضرورت پاکت باز خواهد شد.
سیاست خاتمه مطالعه
این کارآزمایی بالینی تنها در صورت وجود هر یک ازشرایط زیر خاتمه می یابد:
• درخواست کمیته اخلاق برای پایان مطالعه به دلیل بروز مسائل غیراخلاقی
• درخواست محقق اصلی برای پایان مطالعه
تحلیل آماری:
تجزیه و تحلیل آماری دادههای پیامدهای مورد مطالعه با Stata ۱۷.۰ انجام خواهند شد. بررسی نرمال بودن توزیع داده ها با استفاده از آزمون های آماری ()، بررسی نمودارها () و بررسی شاخص های چولگی و کشیدگی بررسی خواهد شد. آنالیز واریانس یک طرفه و آزمون کروسکال-والیس برای مقایسه متغیرهای پارامتریک پیوسته و ناپارامتریک به ترتیب انجام خواهد شد. برای تجزیه و تحلیل نسبت ها از آزمون χ۲ یا Fisher Exact Test استفاده خواهد شد. مقدار پی کمتر از 0.05 به عنوان نقطه برش برای معنی داری استفاده خواهد شد. برای کلیه شرکت کننده هایی که مداخله قطع یا زودتر از موعد از مطالعه خارج می شوند، تمام داده های موجود مرتبط تا زمان قطع در آنالیزها لحاظ می شود. تجزیه و تحلیل میانی پس از جمع‌آوری نیمی از داده‌های شرکت‌کنندگان انجام خواهد شد و مقادیر p < 0.001 از نظر آماری معنی‌دار در نظر گرفته می‌شوند.

**محل انجام مطالعه**

بیمارستان جامع بانوان آرش

**مشخصات ابزار جمع آوری اطلاعات و نحوه جمع آوری**

• از پرسشنامه محقق ساخته که شامل اطلاعات دموگرافیک و بالینی مشارکت کنندگان است، در طول مدت بستری استفاده خواهد شد.
• از visual analogue scale (VAS) برای سنجش شدت درد بیمار در طول مدت بستری استفاده خواهد شد.
• از پرسشنامه ارزیابی زخم بیمار و ناظر (POSAS) برای بررسی وضعیت بهبود زخم بیمار در ویزیت های دوم و سوم (هفته ی اول و چهارم پس از سزارین) استفاده خواهد شد. در رابطه با پرسشنامه POSAS (۱۷) مطالعه های قبلی نشان داده که POSAS می تواند به طور قابل اعتمادی برای ارزیابی اسکارهای پس از جراحی با عکس استفاده شود (۱۸). سایر پرسشنامه ها محقق ساخته هستند.
• از پرسشنامه محقق ساخته ی کمک حافظه شرکت کننده برای درد و مسکن در ویزیت های دوم و سوم (هفته ی اول و چهارم پس از سزارین) استفاده خواهد شد، این پرسشنامه توسط خود بیمار و در مدت زمانی که خارج از بیمارستان است تکمیل می شود.
• پرسشنامه محقق ساخته ی ثبت پیامدهای مورد مطالعه در ویزیت های دوم و سوم (هفته ی اول و چهارم پس از سزارین) استفاده خواهد شد.

**روش محاسبه حجم نمونه و تعداد آن**

با توجه به مطالعه کرومی و همکاران (۱۶) که به بررسی ترمیم و درد زخم سزارین در ۴ روش مختلف بخیه پرداخته اند، حجم نمونه محاسبه خواهد شد. روش متداول برای بخیه ی زخم جراحی سزارین در بیمارستان جامع بانوان آرش استفاده از سوچورهای قابل جذب مونوفیلامان در لایه ی زیرجلد پوست و استفاده از سوچورهای مونوفیلامان نایلونی در خود پوست است. به همین دلیل با توجه به یافته های مطالعه ی مذکور امتیاز قسمت بیمار برای پرسشنامه POSAS برابر ۱۹.۳ (انحراف معیار ۷.۵) و برای قسمت ناظر ۲۰.۶ (انحراف معیار ۷.۶) در نظر گرفته خواهد شد. همچنین میانگین درد برای visual analogue scale (VAS) برابر ۸ (انحراف معیار ۱.۷) در نظر گرفته می شود. با توجه به اعداد مذکور حجم نمونه با در نظر گرفتن آلفای ۵% و توان ۹۰% جهت کاهش ۲۵ درصدی در نمره ی ترمیم زخم و درد زخم بدین صورت محاسبه شد:
با توجه به این که بالاترین حجم نمونه ی بدست آمده عدد ۵۱ بود با در نظر گرفتن ریزش ۱۰ درصدی عدد نهایی برای هر گروه ۵۶ نفر در نظر گرفته شد (در مجموع ۲۲۴ نفر برای چهار گروه).

**محدودیتهای اجرایی طرح و روش کاهش آنها**

عدم تمایل بیماران به شرکت در مطالعه، که با توضیحات لازم توسط محققین و پزشکان به بیماران و ضرورت اجرای مطالعه برطرف می شود. احتمال ریزش افراد به علت عدم تمایل به ادامه شرکت در مطالعه وجود دارد و به همین سبب درصد ریزش در محاسبه حجم نمونه آورده شده است که جایگزین بیمارانی که به هر دلیل تمایل به شرکت ندارند، شود.

جدول زمانبندی

| ردیف | مراحل اجرايی | زمان کل |  |
| --- | --- | --- | --- |
| ۱ | تصویب پروپوزال | ۱ ماه | \| ۱۲ \| ۱۱ \| ۱۰ \| ۹ \| ۸ \| ۷ \| ۶ \| ۵ \| ۴ \| ۳ \| ۲ \| ۱ \| \| --- \| --- \| --- \| --- \| --- \| --- \| --- \| --- \| --- \| --- \| --- \| --- \| |
| ۲ | ثبت در IRCT | ۲ ماه | \| ۱۲ \| ۱۱ \| ۱۰ \| ۹ \| ۸ \| ۷ \| ۶ \| ۵ \| ۴ \| ۳ \| ۲ \| ۱ \| \| --- \| --- \| --- \| --- \| --- \| --- \| --- \| --- \| --- \| --- \| --- \| --- \| |
| ۳ | بیماریابی و اجرای طرح | ۸ ماه | \| ۱۲ \| ۱۱ \| ۱۰ \| ۹ \| ۸ \| ۷ \| ۶ \| ۵ \| ۴ \| ۳ \| ۲ \| ۱ \| \| --- \| --- \| --- \| --- \| --- \| --- \| --- \| --- \| --- \| --- \| --- \| --- \| |
| ۴ | تحلیل و ارائه گزارش نهایی | ۱ ماه | \| ۱۲ \| ۱۱ \| ۱۰ \| ۹ \| ۸ \| ۷ \| ۶ \| ۵ \| ۴ \| ۳ \| ۲ \| ۱ \| \| --- \| --- \| --- \| --- \| --- \| --- \| --- \| --- \| --- \| --- \| --- \| --- \| |

کل مدت زمان اجرا : ۱۲ ماه

هزینه پرسنلی

برای هزینه پرسنلی هیچ داده ای ثبت نشده است.

هزینه وسایل و مواد

برای هزینه وسایل و مواد هیچ داده ای ثبت نشده است.

هزینه آزمایشات و خدمات تخصصی (داخل دانشگاه)

برای هزینه آزمایشات و خدمات تخصصی (داخل دانشگاه) هیچ داده ای ثبت نشده است.

هزینه آزمایشات و خدمات تخصصی (خارج دانشگاه)

برای هزینه آزمایشات و خدمات تخصصی (خارج دانشگاه) هیچ داده ای ثبت نشده است.

هزینه مسافرت

برای هزینه مسافرت هیچ داده ای ثبت نشده است.

هزینه های دیگر

برای هزینه های دیگر هیچ داده ای ثبت نشده است.

نحوه تامین اعتبار طرح

تامین از اعتبارات شخصی مجریان طرح

ملاحظات اخلاقی

**ملاحظات و مشکلات اخلاقی طرح و راه حل های آن**

مطالعه از نوع مداخله ای است و پس از دریافت کد اخلاق پرسشنامه ای از بیماران به منظور رضایت آگاهانه شرکت در مطالعه جمع آوری می شود و بیمارانی که تمایل به شرکت در مطالعه ندارند از مطالعه حذف می شوند. به بیماران اطمینان خاطر داده می شود که اطلاعات شان کاملا محرمانه است و صرفا جهت انجام طرح تحقیقاتی مورد استفاده قرار می گیرد. روش درمانی استاندارد در بیمارستان های دانشگاه علوم پزشکی تهران (من جمله بیمارستان جامع بانوان آرش) پانسمان ساده است. این پانسمان صرفا با استفاده از گاز استریل انجام می شود. مزیت پانسمان ساده همانگونه که از نامش پیداست سادگی آن است ولی معایبی که دارد شامل احتمال بروز عفونت یا باقی ماندن جای زخم پس از ترمیم است. پانسمان ساده هیچ ماده یا عاملی ندارد که باعث پیشگیری از عفونت زخم شود یا این که ترمیم زخم را تسریع کند. مداخلات این مطالعه با فرض این که پرده و مایع باعث کاهش احتمال عفونت و تسریع روند ترمیم زخم خواهند شد، اعمال می شوند. مطالعات گذشته اثر جانبی خاصی را برای استفاده از پرده آمنیون بر روی زخم گزارش نکرده اند. در این مطالعه هیچ کدام از گروه های مداخله و شاهد از درمان استاندارد (پانسمان ساده) محروم نخواهند شد، بلکه مداخلات به صورت ترکیبی از درمان استاندارد + درمان مداخله ای اجرا خواهند شد.
