# Supplementary material for: Effect of autologous amniotic membrane and fluid on wound healing and complications of cesarean section: Study protocol of a factorial randomized controlled trial
Source: PLoS One. 2025 Dec 19;20(12):e0337907. doi: 10.1371/journal.pone.0337907 (PMC12716681; doi:10.1371/journal.pone.0337907)
Supplement: S4 File — The proposal file, which was approved by the IRB translated into English. (DOCX) [file pone.0337907.s004.docx]

**Research Project Title:** Investigating the Effect of Using Autologous Amniotic Membrane and Fluid on Wound Healing and Complications of Cesarean Surgery: A Factorial Randomized Clinical Trial
**Tracking Code:** 69706
**Researcher:** Amene Abiri
**Specialty:** Perinatology Fellowship
**Project Code:** 1402-4-418-69706
**Ethics Code:** IR.TUMS.MEDICINE.REC.1402.758
**Initial Registration Date:** 2023/12/16 – 11:56:22
**Submission Date:** 2024/01/03 – 13:34:59
**Current Version Date:** 2024/02/24 – 14:33:51
**Primary Center:** School of Medicine / Department of Obstetrics and Gynecology (Arash Hospital)
**Secondary Center:** Family Health Research Institute / Mother, Fetus, and Newborn Research Center
**General Information and Abstract of the Proposal**

**Title of the Project:**
Investigating the Effect of Using Autologous Amniotic Membrane and Fluid on Wound Healing and Complications of Cesarean Surgery: A Factorial Randomized Clinical Trial.

**Keywords:**
Wound healing and complications of cesarean surgery, autologous amniotic membrane and fluid

**Type of Study:**
Randomized Clinical Trial

**Summary of the Necessity of the Study:**
Human amniotic membrane and fluid have been used for wound healing (acute and chronic) and burn treatment since the early 20th century. As previously confirmed, their use can reduce healing time, infection, and pain. Studies have shown that the amniotic membrane can induce endothelial cell migration and angiogenesis. A recent meta-analysis demonstrated that patients with chronic lower limb wounds treated with amniotic membrane showed significantly higher healing success over six weeks compared to those with standard care dressings. Furthermore, it has been found that human cryopreserved viable amniotic membrane (hCVAM) can inhibit bacterial growth in wounds, likely due to two antimicrobial peptides, HBD2 and HBD3.

**Summary of Method and Analysis Techniques:**
This study is a prospective, interventional, randomized, controlled, factorial, double-blind clinical trial conducted at Arash Women's Hospital among pregnant women scheduled for cesarean section. The intervention groups are:

- Group A: Dressing with autologous amniotic membrane
- Group B: Dressing with autologous amniotic fluid spray
- Group C: Combination of autologous membrane and fluid spray
- Group D: Standard cesarean wound dressing (control)

Amniotic fluid and membrane are obtained from the patient herself and applied at each wound closure stage. Outcomes will be assessed on postoperative days 2, 10, and 28.

**Is this an international collaborative study?**
No

**Introduction – Problem Statement:**
Cesarean section, widely adopted since James Munro Kerr introduced improved techniques in the early 20th century, has saved many lives. With the increasing number of cesarean procedures, it's now frequently described as an epidemic. Globally, cesarean rates rose from 6.7% in 1990 to 19.1% in 2014. In Iran, the rate is as high as 48%, far exceeding the WHO's recommended 15%, highlighting the importance of addressing associated complications.

The increased preference for cesarean in Iran can be categorized into social-demographic, obstetric-clinical, and non-obstetric-non-clinical factors. Higher education and maternal age are major socio-demographic factors, while prior cesareans and fetal distress are clinical contributors. Non-clinical factors include fear of vaginal birth and physician recommendation. Cesareans offer benefits such as perineal protection, reduced urinary incontinence, reduced fear of labor pain, and quicker recovery.

However, cesareans carry risks such as surgical site infections (SSIs), endometritis, and wound complications like seromas and hematomas, affecting 3–15% of cases. Proper wound dressing significantly influences healing. Negative Pressure Wound Therapy (NPWT) reduces SSI risk but is costly and not cost-effective in low-risk patients.

Amniotic membrane has been historically used for wound healing and burns. It promotes endothelial cell attraction and angiogenesis. Meta-analyses show superior healing in chronic wounds compared to standard care. hCVAM also inhibits bacterial growth via peptides HBD2 and HBD3. Frozen amniotic membranes are now available for wound dressings.

Given the advantages of autologous grafts—lower immune response, reduced disease transmission risk, better outcomes, easier access, lower cost, and fewer ethical concerns—this study investigates the effects of using autologous amniotic membrane and fluid on cesarean wound healing and complications.

**Literature Review:**

- *Mohseni et al.* (2018): In a randomized clinical trial, autologous amniotic membrane dressing reduced post-cesarean pain and analgesic use compared to standard dressing, as measured by VAS.
- *Molazem et al.* (2018): A double-blind trial showed improved wound healing using amniotic membrane, assessed via the REEDA scale.
- *Serena et al.* (2020): A randomized clinical trial on diabetic foot ulcers found significantly higher wound closure rates at weeks 12 and 16 in patients treated with cryopreserved amniotic membrane compared to standard care.

**General, Specific, and Practical Objectives**

**Main Objectives of the Study:**

1. To investigate the effect of autologous amniotic membrane on cesarean wound healing and complications
2. To investigate the effect of sprayed autologous amniotic fluid on cesarean wound healing and complications
3. To assess the effect of simultaneous use of autologous amniotic membrane and sprayed fluid on cesarean wound healing and complications
4. To compare the effects of amniotic membrane, sprayed amniotic fluid, and their combined use on cesarean wound healing and complications

**Specific Objectives of the Study:**

1. To assess the effect of amniotic membrane on wound healing using the POSAS scale on days 10 and 28 post-surgery
2. To evaluate the effect of amniotic membrane on surgical site infection
3. To assess the effect of amniotic membrane on wound dehiscence
4. To assess the effect of amniotic membrane on hematoma formation
5. To assess the effect of amniotic membrane on seroma formation
6. To assess the effect of amniotic membrane on pain at 4, 12, 24, and 36 hours after surgery
7. To assess the effect of amniotic membrane on the frequency of injected analgesic use in the first 36 hours
8. To evaluate the effect of amniotic membrane on the incidence of any adverse events
9. To assess the effect of amniotic membrane on the duration of hospitalization
10. To assess the effect of sprayed amniotic fluid on wound healing using the POSAS scale on days 7 and 28 post-surgery
11. To evaluate the effect of sprayed amniotic fluid on surgical site infection
12. To assess the effect of sprayed amniotic fluid on wound dehiscence
13. To assess the effect of sprayed amniotic fluid on hematoma formation
14. To assess the effect of sprayed amniotic fluid on seroma formation
15. To assess the effect of sprayed amniotic fluid on pain at 4, 12, 24, and 36 hours after surgery
16. To assess the effect of sprayed amniotic fluid on the frequency of injected analgesic use in the first 36 hours
17. To evaluate the effect of sprayed amniotic fluid on the incidence of any adverse events
18. To assess the effect of sprayed amniotic fluid on the duration of hospitalization
19. To assess the combined effect of membrane and fluid on wound healing using the POSAS scale on days 10 and 28
20. To evaluate the combined effect on surgical site infection
21. To assess the combined effect on wound dehiscence
22. To assess the combined effect on hematoma formation
23. To assess the combined effect on seroma formation
24. To assess the combined effect on pain at 4, 12, 24, and 36 hours post-surgery
25. To assess the combined effect on the frequency of injected analgesic use in the first 36 hours
26. To evaluate the combined effect on the incidence of adverse events
27. To assess the combined effect on hospital stay duration
28. To compare the effects of membrane vs. fluid on wound healing (POSAS) on days 10 and 28
29. To compare membrane, fluid, and combination regarding surgical site infection
30. To compare the groups regarding wound dehiscence
31. To compare the groups regarding hematoma formation
32. To compare the groups regarding seroma formation
33. To compare the groups regarding pain at 4, 12, 24, and 36 hours post-surgery
34. To compare the groups regarding injected analgesic use in the first 36 hours
35. To compare the groups regarding adverse events
36. To compare the groups regarding length of hospital stay

**Practical Objective of the Study:**
Using amniotic membrane dressing and sprayed amniotic fluid can potentially reduce common complications of cesarean wounds (pain, infection, hematoma, and scarring). Considering the high cesarean rate among Iranian mothers, any reduction in complications is crucial for improving maternal, neonatal, familial, and community health.

References

۱. Todman D. A history of caesarean section: from ancient world to the modern era. Australian and New Zealand Journal of Obstetrics Gynaecology. ۲۰۰۷;۴۷(۵):۳۵۷-۶۱.

۲. Betrán AP, Ye J, Moller A-B, Zhang J, Gülmezoglu AM, Torloni MR. The increasing trend in caesarean section rates: global, regional and national estimates: ۱۹۹۰-۲۰۱۴. PloS one. ۲۰۱۶;۱۱(۲):e۰۱۴۸۳۴۳.

۳. Rafiei M, Ghare MS, Akbari M, Kiani F, Sayehmiri F, Sayehmiri K, et al. Prevalence, causes, and complications of cesarean delivery in Iran: A systematic review and meta-analysis. International journal of reproductive biomedicine. ۲۰۱۸;۱۶(۴):۲۲۱.

۴. Azami-Aghdash S, Ghojazadeh M, Dehdilani N, Mohammadi M. Prevalence and causes of cesarean section in Iran: systematic review and meta-analysis. Iranian journal of public health. ۲۰۱۴;۴۳(۵):۵۴۵.

۵. Gregory KD, Jackson S, Korst L, Fridman M. Cesarean versus vaginal delivery: whose risks? Whose benefits? American journal of perinatology. ۲۰۱۲;۲۹(۰۱):۰۷-۱۸.

۶. Temming LA, Raghuraman N, Carter EB, Stout MJ, Rampersad RM, Macones GA, et al. Impact of evidence-based interventions on wound complications after cesarean delivery. American journal of obstetrics gynecology. ۲۰۱۷;۲۱۷(۴):۴۴۹. e۱-. e۹.

۷. Conner SN, Verticchio JC, Tuuli MG, Odibo AO, Macones GA, Cahill AG. Maternal obesity and risk of postcesarean wound complications. American journal of perinatology. ۲۰۱۳:۲۹۹-۳۰۴.

۸. Strugala V, Martin R. Meta-Analysis of Comparative Trials Evaluating a Prophylactic Single-Use Negative Pressure Wound Therapy System for the Prevention of Surgical Site Complications. Surgical Infections. ۲۰۱۷;۱۸(۷):۸۱۰-۹.

۹. Shea SK, Soper DE. Prevention of Cesarean Delivery Surgical Site Infections. Obstetrical & Gynecological Survey. ۲۰۱۹;۷۴(۲):۹۹-۱۱۰.

۱۰. McKenna B, Summers NJ. Amnion: The Ideal Scaffold for Treating Full-Thickness Wounds of the Lower Extremity. Clinics in Podiatric Medicine and Surgery. ۲۰۱۸;۳۵(۱):۱-۹.

۱۱. Haugh AM, Witt JG, Hauch A, Darden M, Parker G, Ellsworth WA, et al. Amnion membrane in diabetic foot wounds: a meta-analysis. Plastic and Reconstructive Surgery Global Open. ۲۰۱۷;۵(۴).

۱۲. Mao Y, Hoffman T, Singh-Varma A, Duan-Arnold Y, Moorman M, Danilkovitch A, et al. Antimicrobial peptides secreted from human cryopreserved viable amniotic membrane contribute to its antibacterial activity. Scientific reports. ۲۰۱۷;۷(۱):۱۳۷۲۲.

۱۳. Mohseni F, Saem J, Sekhavati E, Molazem Z, Tabrizi R. Amniotic Membrane for Pain Control After Cesarean Section. Crescent Journal of Medical & Biological Sciences. ۲۰۱۸;۵(۳).

۱۴. Molazem Z, Mohseni F, Rakhshan M, Keshavarzi S, Younesi M. The effect of amniotic membrane on the healing of cesarean wounds: a randomized clinical trial. Women’s Health Bulletin. ۲۰۱۸;۵(۲):۱-۶.

۱۵. Serena TE, Yaakov R, Moore S, Cole W, Coe S, Snyder R, et al. A randomized controlled clinical trial of a hypothermically stored amniotic membrane for use in diabetic foot ulcers. Journal of Comparative Effectiveness Research. ۲۰۲۰;۹(۱):۲۳-۳۴.

۱۶. Cromi A, Ghezzi F, Gottardi A, Cherubino M, Uccella S, Valdatta L. Cosmetic outcomes of various skin closure methods following cesarean delivery: a randomized trial. American journal of obstetrics and gynecology. ۲۰۱۰;۲۰۳(۱):۳۶-e۱.

۱۷. Draaijers LJ, Tempelman FRH, Botman YAM, Tuinebreijer WE, Middelkoop E, Kreis RW, et al. The Patient and Observer Scar Assessment Scale: A Reliable and Feasible Tool for Scar Evaluation. Plastic and Reconstructive Surgery. ۲۰۰۴;۱۱۳(۷).

۱۸. Shao K, Parker JC, Taylor L, Mitra N, Sobanko JF. Reliability of the patient and observer scar assessment scale when used with postsurgical scar photographs. Dermatologic surgery. ۲۰۱۸;۴۴(۱۲):۱۶۵۰.

**Project Team Members**

| **English First Name** | **English Last Name** | **Email** | **Role** | **Specialty** |
| --- | --- | --- | --- | --- |
| Marzieh | Vahid-dastjerdi | [mvahid@tums.ac.ir](mailto:mvahid@tums.ac.ir) | Collaborator | Obstetrics & Gynecology |
| Amene | Abiri | [abiri@sina.tums.ac.ir](mailto:abiri@sina.tums.ac.ir) | Principal Investigator | Perinatology Fellowship |
| Kasra | Jafari | [kasra.e.jafari@gmail.com](mailto:kasra.e.jafari@gmail.com) | Collaborator | Epidemiology |

**Study Execution Method**

**Design:**
This is a prospective, interventional, randomized, controlled, factorial, and double-blind clinical trial.

**Study Location:**
Arash Women's Hospital

**Study Population:**
Pregnant women referred to Arash Hospital for cesarean delivery.

**Inclusion Criteria:**

- Age 18–45 years
- Singleton pregnancy
- Scheduled cesarean delivery
- Gestational age ≥36 weeks at the time of delivery
- Hemoglobin >10 g/dL
- Able to provide informed consent
- Willing to be available throughout the study period and complete all related study steps, including follow-up visits and phone calls

**Exclusion Criteria:**

- Unwillingness to participate
- BMI ≥40 at enrollment
- Placenta previa or placenta accreta
- Previous bowel or urologic surgery
- Multiple gestation
- Known or suspected immunodeficiency (e.g., HIV, Hepatitis B/C)
- Use of immunosuppressive drugs
- Known tobacco or substance use
- Any condition deemed risky by the investigator
- History of keloid formation
- Chorioamnionitis or systemic infections at admission, including subcutaneous infections
- Need for emergency cesarean (e.g., fetal distress, placental abruption, severe preeclampsia/eclampsia)
- Ruptured membranes prior to surgery
- Amniotic fluid stained with meconium or blood
- Multiple vaginal examinations
- Severe preeclampsia with symptoms

**Interventions:**

- **Group A:** Dressing with autologous amniotic membrane
- **Group B:** Dressing with sprayed autologous amniotic fluid
- **Group C:** Combination of both membrane and fluid
- **Group D (Control):** Standard dressing

In Group A, a piece of the patient's own amniotic membrane (sized to the surgical incision) is washed with sterile normal saline and placed on the wound before standard dressing. In Group B, extracted amniotic fluid is sprayed (method detailed below). Group C receives both interventions. Group D receives only standard care dressing.

**Intraoperative Variables Recorded:**

- Time of incision
- Time of uterine incision
- Amniotic fluid description (color, presence of vernix, blood, or meconium)
- Time of uterine closure
- End of surgery
- Estimated blood loss
- Type of skin closure

**Amniotic Fluid Spray Protocol:**

1. After uterine incision, the amniotic sac is opened using an amniotic perforator.
2. A 10-mL syringe collects fluid from the amniotic cavity.
3. The fluid is checked for contamination (e.g., meconium), then attached to the spray device.
4. Fluid is sprayed over the closed uterine incision and lower uterine segment.
5. Fascia is closed, then sprayed with 1.5–2.0 mL of fluid.
6. Subcutaneous tissue and skin are each closed and sprayed with 1.5–2.0 mL.
7. The wound is dressed as usual.

**Follow-Up Schedule:**

1. **Discharge Visit (Day 2–3 post-op):**
   - Evaluation of incision (redness, swelling, firmness, pain at rest and with pressure)
   - Analgesic use since delivery
   - Schedule first follow-up (8–12 days post-op)
   - Review vital signs and document fever (>38°C) and actions taken
   - Record any adverse events
2. **Day 7 Visit (Day 8–12 post-op):**
   - Wound assessment
   - Document signs/symptoms of infection
   - Record adverse events
   - Schedule next visit (4 weeks post-op)
3. **Week 4 Visit:**
   - Wound assessment
   - Document signs/symptoms of infection
   - Record adverse events

**Randomization Method:**
Block randomization with block sizes of 4, 8, and 12 will be used to ensure equal allocation. A random list is generated via Sealed Envelope. Only an external observer responsible for allocation has access to the list. Researchers are blinded and do not participate in allocation. Randomization protocol adherence will be closely monitored.

**Blinding:**
Dressings will appear identical across all groups. Since the membrane is under the dressing, participants remain blinded. Surgical teams cannot be blinded. The physician assessing wound outcomes will be blinded; another clinician will remove the dressing before assessment. Data analysts and follow-up interviewers are also blinded. Only the surgical team and allocation observer are unblinded.

**Unblinding Protocol:**
Unblinding occurs only when medically necessary, such as in the event of a serious adverse event, and only by decision of the PI or co-PI. Group codes are sealed at the study site and opened only in emergencies.

**Study Termination Policy:**
The trial will end if:

- The ethics committee requests termination due to ethical concerns
- The principal investigator decides to terminate the study

**Statistical Analysis:**
Analysis will be performed using Stata 17.0. Normality will be assessed via statistical tests, graphical evaluation, and skewness/kurtosis. One-way ANOVA and Kruskal-Wallis tests will be used for parametric and non-parametric continuous variables, respectively. Chi-square or Fisher’s Exact tests will be used for proportions. A p-value <0.05 is considered statistically significant. For participants who discontinue or are lost to follow-up, available data will be included. Interim analysis will be performed at 50% enrollment, with p < 0.001 considered significant.

**Study Location:**
Arash Women's Hospital

**Data Collection Tools and Methods:**

- A researcher-designed questionnaire will be used during hospitalization to collect demographic and clinical information.
- The Visual Analogue Scale (VAS) will be used to assess the intensity of pain during hospitalization.
- The Patient and Observer Scar Assessment Scale (POSAS) will be used in the second and third follow-up visits (weeks 1 and 4 post-cesarean) to evaluate wound healing. Previous studies have validated POSAS for postoperative scar assessment using photographs. Other questionnaires are researcher-designed.
- A participant memory aid questionnaire for pain and analgesic use will be completed by the patient at home and reviewed during the second and third visits.
- A researcher-designed outcome recording form will be used during the second and third follow-up visits.

**Sample Size Calculation:**
Based on the study by Cromi et al. (16), which compared cesarean wound healing and pain across four suturing techniques, the following assumptions were made:

- POSAS patient score mean = 19.3 (SD = 7.5)
- POSAS observer score mean = 20.6 (SD = 7.6)
- VAS mean pain score = 8 (SD = 1.7)
  With a significance level (alpha) of 5% and power of 90% to detect a 25% reduction in wound healing and pain scores, the highest calculated required sample size was 51 per group. Allowing for a 10% attrition rate, the final sample size is set at 56 participants per group, totaling 224 participants across four groups.

**Operational Limitations and Mitigation Strategies:**
A potential limitation is participants' unwillingness to join the study. This will be addressed through proper explanation by researchers and physicians about the importance of the study. Anticipated dropout has been accounted for in the sample size calculation to ensure adequate final enrollment.

**Timeline Table**

| **Row** | **Implementation Stage** | **Total Duration** |
| --- | --- | --- |
| 1 | Proposal approval | 1 month |
| 2 | Registration in IRCT | 2 months |
| 3 | Patient recruitment and study execution | 8 months |
| 4 | Analysis and final report presentation | 1 month |

**Total implementation duration: 12 months**

**Personnel Costs**
No data recorded for personnel costs.

**Equipment and Materials Costs**
No data recorded for equipment and materials costs.

**Costs for Tests and Specialized Services (Inside University)**
No data recorded.

**Costs for Tests and Specialized Services (Outside University)**
No data recorded.

**Travel Costs**
No data recorded.

**Other Costs**
No data recorded.

**Funding Source for the Project**
Funded by the personal credits of the project executors.

**Ethical Considerations:**

This is an interventional study, and after obtaining the ethical approval code, a questionnaire will be collected from patients for informed consent to participate in the study. Patients unwilling to participate will be excluded. Patients will be assured that their information is completely confidential and used solely for the research project.

The standard treatment method in Tehran University of Medical Sciences hospitals (including Arash Women's Hospital) is simple dressing, which only uses sterile gauze. The advantage of simple dressing, as the name implies, is its simplicity, but disadvantages include the possibility of infection or residual scar after healing. Simple dressing contains no agent that prevents wound infection or accelerates wound healing.

The interventions in this study are applied based on the hypothesis that the membrane and fluid will reduce the probability of infection and speed up wound healing. Previous studies have reported no specific side effects for the use of amniotic membrane on wounds.

In this study, none of the intervention or control groups will be deprived of standard treatment (simple dressing). Instead, interventions will be combined with standard treatment + interventional treatment.
