# Supplementary material for: Effect of autologous amniotic membrane and fluid on wound healing and complications of cesarean section: Study protocol of a factorial randomized controlled trial
Source: PLoS One. 2025 Dec 19;20(12):e0337907. doi: 10.1371/journal.pone.0337907 (PMC12716681; doi:10.1371/journal.pone.0337907)
Supplement: Attachment — Submitted filename: Response 2025-10-06.pdf [file pone.0337907.s008.pdf]

Dear Dr. Ahmed Mohamed Maged,

We sincerely appreciate the time and effort you and the reviewer have dedicated to evaluating our manuscript, "Effect of Autologous Amniotic Membrane and Fluid on Wound Healing and Complications of Cesarean Section: Study Protocol of A Factorial Randomized Controlled Trial" (Manuscript ID: PONE-D-25-30795R2). We are grateful for the constructive feedback, which has helped us improve the quality of our work.

We have carefully addressed all the comments and suggestions provided by the reviewer. Below, we provide a point-by-point response to each remark, detailing the revisions made in the revised manuscript. All changes have been highlighted in the revised version for easy reference in yellow.

Reviewer #1:

We sincerely thank you for your insightful and detailed comments.

Comment 1: *"Please mention the primary outcomes and how did you measure all outcomes? What are the statistical tests?"*

Response 1: As suggested, in the "Abstract" we have now stated the primary outcome of the study and detailed the measurement methods for all outcomes, along with the statistical tests used (lines 40-43).

Comment 2: *"All of benefits that authors mentioned about cesaean section in comparison to normal vaginal delivery, in fact are the hazards of this surgery such as: shorter hospitalization, shorter recovery, better breastfeeding. Please revise."*

Response 2: As requested, we removed the "shorter hospitalization, shorter recovery, and better breastfeeding" (lines 62-63).

Comment 3: *"Lines 102-103 needs to be cited."*

Response 3: As per your request, we added relevant citations to support the claims made (lines 104).

Comment 4: *"Line 95: please write the reference."*

Response 4: We thank the reviewer for raising this point. This sentence is a concluding statement based on the evidence and reasoning presented earlier in our introduction, where we detail the high cost of processed allografts (lines 86-88), the risk of immunological rejection (lines 88-91), and the logistical simplicity of using autologous tissue available at the point-of-care (lines 87-88). However, to acknowledge the reviewer's valid point that this is a synthesizing claim, we have tempered the language to more clearly present it as our study's conclusion. The revised sentence now reads:

"Based on the factors outlined above, we conclude that in the context of cesarean deliveries, autologous AM may represent a cost-effective, immunologically compatible, and clinically practical alternative to processed grafts." (lines 94-96).

Comment 5: *"What was the minimum and maximum score of POSAS? Is there any cut-off?"*

Response 5: As per your request, we provided information about the minimum and maximum scores of POSAS and cut-off values. (lines 147-150).

Comment 6: *"Statistical analyses: did authors attempt to report effect size?"*

Response 6: Since this is a protocol of a study whose results are not ready yet, we cannot report the effect sizes currently. But after the results are ready effect sizes of two-way ANOVA (mean±SD) and logistic regression (odds ratio) will be presented.

Comment 7: *"Line 335: why authors wrote that human ethics and consent to participate is NA?"*

Response 7: Thank you for raising this issue. We corrected and added an ethical declaration to this section (lines 359-364).

Reviewer #2

We are truly grateful for your kind and encouraging words. Your positive feedback means a great deal to our team and reinforces our commitment to further research in this area. Thank you for taking the time to review our work so thoughtfully.

Reviewer #3

Thank you for taking the time to review our work. We're grateful for your guidance throughout the review process.

Comment 1: *"Thank you for your updated protocol - please note however, that given the breakdown that is given in the response it is possible to unblind the statistician as for ghroups A,B,C,D you are telling us precisely which comparison they are in. So please remove the statement at line 252 as it is not correct."*

Response 1: We removed that statement.

Reviewer #4

We sincerely thank you for your time and valuable feedback.

Comment 1: *"The references in the text need to be updated. The manuscript should be re-edited"*

Response 1: We have carefully considered all comments and have undertaken a comprehensive revision of the manuscript to address them. We used Grammarly to improve the writing of our manuscript. Also, we added some recently published citations.

Reviewer #5

Thank you for taking the time to share your valuable feedback with us.

Comment 1: *"Line 54: The phrase "… CS increased by 12.4% (from 6.7% to 19.1%)" should be edited. First, CS should be spelled out. Second, increased by 12.4% implies a relative increase (RRI), whereas the authors mean an absolute increase (ARI)."*

Response 1: We revised the sentence, and it now reads (line 56):

"In the last three decades, CS increased from 6.7% to 19.1% globally."

Comment 2: *"Lines 59–61: The reference used for the sentence is out of date (2012), and I think some of the mentioned advantages (for example, shorter recovery, shorter length of hospitalization, more successful breastfeeding, and control over the birth process) are not correct."*

Response 2: We removed the mentioned advantages (shorter recovery, shorter length of hospitalization, more successful breastfeeding, and control over the birth process) and updated the reference to a meta-analysis published in 2023 (reference No. 6). (lines 62-63).

Comment 3: *"The expected recruitment start date registered in the IRCT (2024-07-22) differs from what is reported in the manuscript as the recruitment start date (01/10/2024). Please describe the reason in the protocol."*

Response 3: The reason for the difference is a logistical delay in the initiation of the study. The date registered in the IRCT (2024-07-22) was the projected start date at the time of registration. However, the completion of site preparation tasks was finalized later than anticipated. Therefore, the actual recruitment of the first participant began on 01/10/2024, as reported in the manuscript. To ensure transparency and accuracy, we have added a brief explanation in the 'Methods' section of the manuscript to clarify this. The added text reads:

"While the initial registration in the IRCT indicated a projected start date of 22/07/2024, the actual recruitment of participants began on 01/10/2024 due to administrative finalizations. " (lines 114-116).

Comment 4: *"Line 35: It is mentioned that the trial is double-blind, but the person performing the intervention could not be blinded. According to CONSORT, please specify in the abstract which groups are blinded."*

Response 4: We revised the abstract and added a brief explanation of the blinded parties (lines 35-36).

Comment 5: *"It is better not to use uncommon abbreviations such as "PO.""*

Response 5: We revised the text and replaced "PO" with "Postoperative".

Comment 6: *"Table 1: It is suggested to use "×" for each assessment time point instead of the current symbol."*

Response 6: We revised Table 1 and replaced "×" with "X".

Comment 7: *"Please report which number was used for the numerator degrees of freedom. I used 3, and the calculated sample size was 141."*

Response 7: We used df = 1 for the calculation. In a 2×2 factorial trial, each effect of interest (main effect of AM, main effect of AF, and their interaction) compares two levels. The numerator df is therefore 1 for each test (AM: 2–1=1; AF: 2–1=1; AM×AF: (2–1)(2–1)=1).

The df = 3 applies only to an overall 4-group ANOVA (testing any difference among the four means), but in a factorial design, the analysis is based on main effects and interaction contrasts,

each with df = 1. That is why we used df = 1 in the power calculation and added it in the text (line 293).

Comment 8: *"Most of the outcomes will be measured more than once. Please explain how time and the interaction of time × group will be assessed during analysis."*

Response 8: We thank you for this important comment and for emphasizing the need to clarify how time and the time×group interaction will be handled in the analyses.

In this study, outcomes are measured at multiple time points for different practical and clinical reasons, and the importance of "time" varies by outcome. The primary outcome, POSAS, will be analyzed at day 28, which represents the final cosmetic and functional quality of wound healing. The day-10 measurement is collected because patients routinely attend for suture removal at that visit, providing an opportunity to capture early healing data and reduce potential loss to follow-up if some participants do not return at day 28. Since there are only two time points, a longitudinal model would have limited statistical power and add little additional information beyond the final outcome. Therefore, the main analysis will compare POSAS scores at day 28 using two-way ANOVA with factors AM, AF, and AM×AF.

For surgical site infection (SSI), repeated assessments at 36 hours, day 10, and day 28 are intended solely to detect any incident infection within the postoperative period. The timing of onset is not clinically central, and the main parameter of interest is the presence or absence of infection within 28 days. Accordingly, SSI will be analyzed as a binary outcome using logistic regression.

For pain (VAS), which is dynamic and clinically influenced by time, we will conduct a linear mixed-effects model (LMM) including fixed effects for time, AM, AF, and their interactions. If the sample size proves insufficient for stable model estimation, we will instead present two-way ANOVA results at each time point. These clarifications and justifications have been incorporated into the revised Statistical Analysis section.

We added the necessary amendments to the "Statistical Analysis" section (lines 305-326).

Comment 9: *"Please describe where all data underlying the findings will be made available when the study is complete."*

Response 9: We revised the "Availability of data and materials" declaration (lines 368-370).

Sincerely,

Dr. Amene Abiri
