# Supplementary material for: Effect of autologous amniotic membrane and fluid on wound healing and complications of cesarean section: Study protocol of a factorial randomized controlled trial
Source: PLoS One. 2025 Dec 19;20(12):e0337907. doi: 10.1371/journal.pone.0337907 (PMC12716681; doi:10.1371/journal.pone.0337907)
Supplement: Attachment — Submitted filename: Response 2025-08-10.docx [file pone.0337907.s006.docx]

**Dear Dr. Ahmed Mohamed Maged,**

We sincerely appreciate the time and effort you and the reviewers have dedicated to evaluating our manuscript, "Effect of Autologous Amniotic Membrane and Fluid on Wound Healing and Complications of Cesarean Section: Study Protocol of A Factorial Randomized Controlled Trial" (Manuscript ID: PONE-D-25-30795). We are grateful for the constructive feedback, which has helped us improve the quality of our work.

We have carefully addressed all the comments and suggestions provided by the reviewers. Below, we provide a point-by-point response to each remark, detailing the revisions made in the revised manuscript. All changes have been highlighted in the revised version for easy reference by yellow color.

Editor:

1. Journal Style Requirements:

We have carefully reviewed and ensured that the manuscript now fully complies with PLOS ONE’s style and formatting requirements.

2. ORCID iD for Corresponding Author:

As requested, we have added the ORCID iD of the corresponding author to the submission portal.

3. Ethics Statement:

The ethics statement has been moved to the Methods section only, as per the journal’s guidelines.

4. Data Availability Statement Amendments:

We have revised the Data Availability Statement to clarify that this is a study protocol and does not involve datasets for sharing at this stage. The updated statement now reads:

"This is a study protocol, and no datasets were generated or analyzed during the current study. Data sharing is not applicable to this article."

5. Author Affiliations:

We have double-checked and confirmed the accuracy of each author’s affiliation.

6. Removal of personal information from the dataset.

Since it is a study protocol it does not involve datasets for sharing at this stage.

7. Table 1 Placement:

Table 1 is now included within the main manuscript text (Lines 180).

8. Supporting Files:

The supporting files submitted (e.g., ethical approval letter) are those required by the editorial office and will not be published with the manuscript.

**Reviewer #1:**

We sincerely thank you for your thoughtful and constructive feedback. Your comments helped us clarify important aspects of the study and improve the overall quality of the manuscript.

**Comment 1 Background:** Starting the background directly from CS and its complications.

**Response 1:** We removed the text about childbirth and CS history and now the background begins from the CS and its complications directly.

**Comment 2 Background:** Redundant text about why women choose CS.

**Response:** Thanks. We just removed the redundant text.

**Comment 3 Background:** Add quantitative data on AM’s efficacy.

**Response 3:** We added the requested data (lines 73-75), thanks.

**Comment 4 Background:** clarify why autologous amniotic membrane and fluid outperform processed products.

**Response 4:** We have expanded our introduction section to explicitly compare autologous and processed AM and AF, specifically addressing cost and immunogenicity (lines 82-95).

**Comment 5 Background:** Advantages and disadvantages of CS.

**Response 5:** We just removed this part, thanks.

**Comment 6 Background:** Dehiscence typo.

**Response 6:** It was a typo and we corrected it (line 65).

**Comment 1 Methods:** "As the researchers are not blind, this study is not double blind, and this is single blind."

**Response 1:** We respectfully clarify that our study design does indeed meet the criteria for a double-blind trial according to the standard definition provided in *Fundamentals of Clinical Trials* (Friedman et al., 4th edition). As explicitly stated on page 151:

*"In a double-blind study, neither the participants nor the investigators...responsible for following the participants, collecting data, and assessing outcomes should know the identity of the intervention assignment."*

Crucially, the text further specifies (page 152):

*"It is theoretically possible to design a study comparing two surgical procedures...in which the surgeon performing the operation knows...but neither the study investigator nor the participant knows."*

In our trial:

- The operating surgeons (who necessarily know the intervention during the procedure) have **NO** role in post-operative assessment, data collection, or outcome evaluation.
- Participants are blinded to their treatment allocation.
- Assessing investigators (responsible for follow-up, data collection, and endpoint adjudication) remain fully blinded.

This configuration satisfies the double-blind criteria as:

- The knowledge of the performing surgeons does not compromise blinding of either participants or outcome assessors.
- Only those requiring procedural awareness are unblinded, while all other study personnel maintain blinding.

**Comment 2 Methods:** "Authors should explain the POSAS scale in more detail. How many questions does it have? Who validated this scale in Iran?"

**Response 2:** We have revised the manuscript to include a detailed description of the POSAS 3.0, specifying the number of items on the Patient Scale, and added information on its validated Persian version by Naghizadeh et al. Please see the updated Methods section for these additions. Thanks

**Comment 3 Methods:** "Are the patients hospitalizing until 36 hours after C/S, if not, how authors evaluate the pain of participants in this time?"

**Response 3:** Yes, at our center, patients typically stay 36 hours post-operative.

**Comment 4 Methods:** "Add screening flowchart (CONSORT-style) showing enrollment numbers at each stage"

**Response 4:** As this is a study protocol, final enrollment data are not yet available. However, in line with CONSORT recommendations for protocols, there is a planned participant flow diagram illustrating the anticipated stages of recruitment, allocation, and follow-up under **Table 1**.

**Comment 5 Methods:** "Specify who conducts baseline assessments (trained nurses? residents?)"

**Response 5:** We added on line 192 that trained medical residents will conduct the base assessments.

**Comment 6 Methods:** "Line 159: Why authors consider gestational age of 36 weeks, that is not term pregnancy."

**Response 6:** We have revised the text to clarify that we are referring to completed 36 weeks (at least completed 36 weeks (≥37+0 weeks)) of gestation. This correction has been made in the manuscript on line 196.

**Comment 7 Methods:** "Line 160: Hb: 10g/dL is considered anemia and it can deteriorate wound healing."

**Response 7:** We agree that hemoglobin levels below 10 g/dL may impair wound healing. For this reason, we only included Hb **above** the 10 g/dL in our study, as mentioned in line 197. In the Iranian pregnant population, the definition of normal hemoglobin is as … (ref please).

**Comment 8 Methods:** "A randomized list is generated using the sealed envelope is not correct and needs revision."

**Response 8:** As we clarified in the manuscript, the randomization list was generated using the Sealed Envelope website, an online tool specifically designed for generating randomization sequences (link: <https://www.sealedenvelope.com/>).

**Comment 9 Methods:** "Line 220: the explanation of allocation concealment is not enough."

**Response 9:** We modified the text to explain concealment in more detail (lines 261-265).

**Comment 10 Methods:** "Please explain Storage duration/temperature for AM/AF and standardized wound closure technique across groups."

**Response 10:** We modified the text to explain Storage duration/temperature in more detail (lines 218-229).

**Comment 11 Methods:** "Add Safety: Add protocol for adverse event reporting (e.g., allergic reactions)."

**Response 11:** We modified the text and added a subsction titled “Safety and adverse event reporting” as per your request (lines 170-177).

**Comment 1 Discussion:** "Comparative Context: Contrast with similar AM studies in non-CS wounds”

**Response 1:** To the best of our knowledge, no previous study has investigated the combined or separate application of autologous AM and AF specifically on cesarean section or other surgical wounds. Most existing research on AM has focused on chronic wounds such as diabetic foot ulcers, pressure ulcers, and burn injuries, which differ fundamentally from surgical wounds in terms of pathophysiology, healing dynamics, and risk factors. Therefore, direct comparisons are limited, and our study contributes novel insights by evaluating the potential regenerative effects of AM and AF in the context of acute, surgically induced tissue injury.

**Comment 2 Discussion:** "Implementation: Address feasibility of AM/AF use in low-resource settings.”

**Response 2:** Thanks a lot. We modified the text and added a paragraph discussing feasibility of AM/AF use in low-resource settings (lines 320-327).

**Comment 3 Discussion:** "Limitations: Acknowledge single-center design and short follow-up.”

**Response 3:** We modified the text and added the limitations of the protocol as per your request (lines 315-318).

**Reviewer #2:**

We are truly grateful for your kind and encouraging words. Your positive feedback means a great deal to our team and reinforces our commitment to further research in this area. Thank you for taking the time to review our work so thoughtfully.

**No comments needing edits**

**Reviewer #3:**

We sincerely thank you for your insightful and detailed comments, particularly regarding the methodological aspects of the study. Your suggestions were very important in strengthening our methods section, and we greatly appreciate the time and expertise you dedicated to improving the quality of our manuscript.

**Comment 1:** "The manuscript needs a clearer justification of the use of both AM and AF in the same study. Given their similar biological properties, explain why both were included, and whether any previous studies have used these interventions for surgical wounds such as cesarean incisions.”

**Response 1:** While AM and AF both promote wound healing, they differ in important ways. AM provides a physical barrier and delivers structural support along with anti-inflammatory and growth-promoting factors, while AF is a fluid containing stem cells and soluble signaling molecules that act more through paracrine mechanisms. These differences suggest potential complementary effects. To the best of our knowledge, no prior studies have evaluated the use of AM and AF alone or in combination for surgical wounds such as cesarean incisions. Previous research has primarily focused on chronic wounds (e.g., diabetic ulcers, pressure ulcers, burns) and has used donor-derived products, which are costly and not always feasible in surgical settings. In contrast, our study used autologous AM and AF, collected and applied during cesarean delivery, making the intervention both practical and cost-effective. We have clarified this rationale in the Introduction section (lines 96-103).

**Comment 2:** "It would be better to describe this as a 2x2 design and not a four arm study of 3 interventions and 1 control - there are two interventions, each of which is given or withheld.”

**Response 2:** Thank you so much. We modified the text and corrected the study designs as a 2x2 factorial design as per your request (lines 109-112).

**Comment 3:** "The interventions do not sound blinded - how are the sprays applied, and should one not have a dummy spray - the preparation of the interventions seems to be different. How is one to avoid performacne bias in the period before the assessment by a blinded observer.”

**Response 3:** We acknowledge the challenge of blinding in this study due to the physical nature of the interventions (membrane application and fluid spray), which made blinding of the surgical team impractical. However, both patients and outcome assessors are blinded to group allocation to minimize detection bias. We have now clarified this in the manuscript and acknowledged the potential for performance bias as a limitation (lines 318-319).

**Comment 4:** "I think the statistician needs to be aware of allocation (line 211) to analyse the data.”

**Response 4:** To minimize bias during data analysis, the statistician will receive a masked dataset with group labels (A, B, C, D) without knowledge of which label corresponds to which intervention. This will allow for a blinded analysis of outcomes. Group identities will only unblinded after all statistical comparisons are completed.

**Comment 5:** "There is insufficient guarding of allocation concealment given the use of blocking in a single centre. Closed envelopes do not provide good concealment (see the excellent example in Altman's book on medical statistics), so how will this be policed?”

**Response 5:** We have revised the manuscript to provide more detail on our allocation concealment process (lines 261-265). As described, we use sequentially numbered, opaque, sealed envelopes, stored securely and managed by an independent research assistant not involved in recruitment or assessment. While we recognize that central randomization or other electronic systems may offer stronger concealment, such methods are not feasible in our setting due to resource limitations.

**Comment 6:** "If the pain on a VAS is 8 with sd 1.7 it is not normally distributed - how was this taken into account in the sample size calculation.”

**Response 6:** Initially, we used the two-sample t-test formula to calculate the sample size, as it is a standard and conservative approach. It is well-documented that t-test–based calculations tend to slightly overestimate the required sample size in nonparametric settings, thus providing a margin of safety in study planning. However, based on your suggestion, we have also included a nonparametric sample size calculation using Noether’s method for the Mann–Whitney U test, which confirms that our study is adequately powered to detect a clinically meaningful difference in VAS pain scores.

We have now added this explanation and citation to the revised manuscript (lines 279-285).

**Comment 7:** "To allow for 10% dropout one must inflate by 1/(0.9) - so the sample size would need to be slightly more than 56 - as it is 56 does not allow for 10% dropout but rather only about 9%.”

**Response 7:** Thanks. You are right. We just corrected the sample size to 57 for each group.

**Comment 8:** "Also this is two 2-way comparisons - so if one wants to power each intervention on the differences seen you need only 104 patients in total in each comparison. In other words the sample size is out by a factor of two since every patient in a factorial design counts double.”

**Response 8:** You are absolutely right that in a 2×2 factorial design, assuming no interaction between the interventions, each participant contributes to the analysis of both main effects (AM vs no AM and AF vs no AF). This efficiency allows for fewer total participants compared to a traditional four-arm trial.

Our original sample size was calculated conservatively based on individual group comparisons, without initially accounting for the statistical efficiency of the factorial design. While our design allows for main effect estimation using fewer participants, we opted for a larger sample size to also allow for exploratory analysis of potential interaction effects between AM and AF (if needed), and to maintain sufficient power in case of unexpected dropout or deviations from the additivity assumption.

**Comment 9:** "The analyses are not described in suffiicent detail or in a fashion appropriate to a factorially designed trial.”

**Response 9:** Thanks. We have revised the statistical analysis section to appropriately reflect the 2×2 factorial design and included details on main effects, interaction testing, and analysis methods for each outcome (lines 292-304).

**Sincerely,**

**Dr. Amene Abiri**
