# Supplementary material for: Effect of autologous amniotic membrane and fluid on wound healing and complications of cesarean section: Study protocol of a factorial randomized controlled trial
Source: PLoS One. 2025 Dec 19;20(12):e0337907. doi: 10.1371/journal.pone.0337907 (PMC12716681; doi:10.1371/journal.pone.0337907)
Supplement: Attachment — Submitted filename: Response 2025-09-08.docx [file pone.0337907.s007.docx]

**Dear Dr. Ahmed Mohamed Maged,**

We sincerely appreciate the time and effort you and the reviewer have dedicated to evaluating our manuscript, "Effect of Autologous Amniotic Membrane and Fluid on Wound Healing and Complications of Cesarean Section: Study Protocol of A Factorial Randomized Controlled Trial" (Manuscript ID: PONE-D-25-30795). We are grateful for the constructive feedback, which has helped us improve the quality of our work.

We have carefully addressed all the comments and suggestions provided by the reviewer. Below, we provide a point-by-point response to each remark, detailing the revisions made in the revised manuscript. All changes have been highlighted in the revised version for easy reference by yellow color.

**Reviewer #3:**

We sincerely thank you for your insightful and detailed comments. Your suggestions were very important in strengthening our methods section, and we greatly appreciate the time and expertise you dedicated to improving the quality of our manuscript.

In case if you believe that our amnedments do not fully satisfy your comments, please provide us with the exact sample size calculation formula (or software) and any specific statistical analysis technique you consider suitable for our methodology.

**Comment 1:** *"I note now that this is billed as a factorial design - so the power is different, and the calculation needs to show what the actual power is here for the design as chosen.”*

**Response 1:** Thank you for your detailed comment. We acknowledge the need to present the sample size in the context of a 2×2 factorial design. We have recalculated and clarified the sample size justification for the factorial design and added the full calculations to the revised manuscript (see revised Sample Size subsection). Briefly, the study has two factors (AM: present/absent; AF: present/absent). We powered the trial to detect a clinically meaningful 25% reduction in the POSAS patient score (same assumption used previously) (lines 272-290).

**Comment 2:** *"Please note also that you cannot analyse a factorial design given A vs B vs C vs D - it is okay not to know what is treatment and placebo (ie block blinding, not true blinding) but the two interventions need to be given separately to allow for the analyses proposed.”*

**Response 2:** Thank you so much. We agree that the statistical analysis must be factorial (main effects and interaction) rather than a simple A vs B vs C vs D comparison. We have revised the Methods to state explicitly that the main effects will be estimated by pooling across the relevant cells (AM present = groups A+C vs AM absent = groups B+D; AF present = B+C vs A+D), and that the AM × AF interaction will be tested by inclusion of the interaction term. We will report main effects and interaction statistics (with pairwise comparisons only if the interaction is significant) (lines 294-297 and 303-305).

**Sincerely,**

**Dr. Amene Abiri**
